# A Cross-Species Transmission of a Camel-Derived Genotype 8 Hepatitis E Virus to Rabbits

**DOI:** 10.3390/pathogens10111374

**Published:** 2021-10-24

**Authors:** Wenjing Zhang, Yasushi Ami, Yuriko Suzaki, Michiyo Kataoka, Naokazu Takeda, Masamichi Muramatsu, Tiancheng Li

**Affiliations:** 1 Department of Virology II, National Institute of Infectious Diseases, Tokyo 208-0011, Japan; zwjviolin@foxmail.com (W.Z.); muramatsu@nih.go.jp (M.M.); 2 Division of Experimental Animals Research, National Institute of Infectious Diseases, Tokyo 208-0011, Japan; yami@nih.go.jp (Y.A.); ysuzaki@nih.go.jp (Y.S.); 3 Department of Pathology, National Institute of Infectious Diseases, Tokyo 208-0011, Japan; michiyo@nih.go.jp; 4 Research Institute for Microbial Diseases, Osaka University, Osaka 565-0781, Japan; seishunaotake@gmail.com

**Keywords:** bactrian camel hepatitis E virus, HEV-5, HEV-7, HEV-8, cross-species infection, rabbit, virus-like particle, VLP

## Abstract

Novel genotypes of hepatitis E virus (HEV), i.e., HEV-5, HEV-7, and HEV-8, have been identified in wild boar, dromedary camels, and Bactrian camels, respectively, and they transmit to cynomolgus monkeys in a trans-species manner, raising the potential for zoonotic infection. Rabbits are the natural reservoir for rabbit HEV, but they are also susceptible to HEV-3 and HEV-4. It has been unknown whether rabbits are susceptible to HEV-5, HEV-7, and HEV-8. To investigate the infectivity of novel HEVs in rabbits and to assess whether rabbits are appropriate animal models for these HEVs, we inoculated Japanese white rabbits with HEV-5, HEV-7, and HEV-8, respectively. We observed that viral RNA was present in the fecal specimens of the HEV-8-inoculated rabbits and anti-HEV IgG antibodies were present in its sera, although anti-HEV IgM was undetectable and no significant elevation of ALT was observed. These results indicated that HEV-8 crossed species and infected the rabbits. No evidence for replication was observed in HEV-5 and HEV-7, suggesting that rabbits are not susceptible to these genotypes. The antibodies elicited in the HEV-8-infected rabbits did not protect them from the rabbit HEV challenge, suggesting that the antigenicity differs between HEV-8 and rabbit HEV. Antigenic analyses demonstrated that anti-HEV-8 antibodies reacted more strongly with homologous HEV-8 virus-like particles (VLPs) compared to heterologous rabbit HEV VLPs, but anti-rabbit HEV antibody had similar reactivity to the VLPs of rabbit HEV and HEV-8, suggesting that HEV-8 lacks some epitope(s) that exist in rabbit HEV and induced the neutralizing antibodies against rabbit HEV.

## 1. Introduction

Hepatitis E virus (HEV) is a causative agent of acute and chronic hepatitis E in humans and is distributed worldwide [1,2]. HEVs are classified into the family *Hepeviridae*, which is divided into two genera, *Orthohepevirus* and *Piscihepevirus* [3]. The genus *Orthohepevirus* includes at least four species: *Orthohepeviruses A* to *D* [3]. The species *Orthohepevirus A* includes eight genotypes designated genotypes 1 to 8 (HEV-1 to HEV-8) [3,4,5]. Although most cases of hepatitis E have been caused by HEV-1 to HEV-4, novel HEVs including HEV-5, HEV-7, and HEV-8 are known to cross-transmit to non-human primates such as cynomolgus monkeys, raising a potential risk of zoonotic infection [6,7,8,9]. In fact, a human case of hepatitis E due to HEV-7 infection was reported in 2016 [10].

HEV-5, HEV-7, and HEV-8 were first detected in wild boar, dromedary camels, and Bactrian camels, respectively [11,12,13]. Since replication-competent strains of these viruses were successfully produced by a reverse genetics system using a human hepatocarcinoma cell line, PLC/PRF/5 [6,7,9], the mechanism of the replication of these HEVs might be elucidated using the cell culture ex vivo. The original reservoirs of HEV-5, HEV-7, and HEV-8 are large animals that are not easily used as animal models, especially to understand the pathogenicity of HEV. There is an urgent need for an appropriate small-animal model for the study of the new HEVs.

Cynomolgus monkeys are susceptible to HEV-5, HEV-7, and HEV-8, but the costs accompanying their use are high, and recent studies indicated that most of the imported cynomolgus monkeys have been exposed to HEV infection, suggesting that the source of the monkey is limited [14]. Our research has confirmed that rats and mice are not susceptible to HEV-8 infection [9]. A tractable small animal remains necessary for this line of research.

Rabbit HEV was isolated from rabbits and assigned to HEV-3ra, a subtype of HEV-3 [4,15]. Rabbits are the original reservoir of rabbit HEV; they are known to be susceptible to HEV-3 and HEV-4, and they are used as a small-animal model in infection experiments [16,17]. In the present study we inoculated rabbits with HEV-5, HEV-7, and HEV-8 to investigate whether the rabbits are susceptible to these genotypes.

## 2. Materials and Methods

### 2.1. HEV Strains

HEV-5 (AB573435), HEV-7 (KJ496144), HEV-8 (MH410176), and rabbit HEV (LC484431) were used in this study [8,11,12,17]. All of these HEV strains were produced by a reverse genetics system using a human hepatocarcinoma cell line, PLC/PRF/5 cells (JCRB0406) (JCRB Cell Bank, Osaka, Japan), containing both “quasi-enveloped viruses” and “naked viruses” and that induced infection in cynomolgus monkeys or rabbits. [6,7,9,17]. The culture supernatants of the HEV-infected PLC/PRF/5 cells were clarified by centrifugation at 10,000× *g* for 30 min and then passed through a 0.45 µm membrane filter (Millipore, Bedford, MA, USA). The copy numbers of the viral RNA were adjusted to 10^7^ copies/mL and stored at −80 °C until use. Infectivity of the stock viruses were reconfirmed by inoculating PLC/PRF/5 cells after inoculation of the animals.

### 2.2. Inoculation of Rabbits and the Sample Collection

Nine 8-month-old female Japanese white rabbits (SLC, Hamamatsu, Japan) were used to examine the cross-species transmission of HEV-5, HEV-7, and HEV-8. The rabbits were separated into three groups and then intravenously inoculated with 1 mL of the HEV (10^7^ copies/mL) through an ear vein. Rabbits G5Rb-1, G5Rb-2, and G5Rb-3 were inoculated with the HEV-5. Rabbits G7Rb-1, G7Rb-2, and G7Rb-3 were inoculated with the HEV-7. Rabbits G8Rb-1, G8Rb-2, and G8Rb-3 were inoculated with the HEV-8. Serum samples were collected from the rabbits weekly and used for the detection of the RNA, the anti-HEV IgG and IgM antibodies, and the alanine aminotransferase (ALT) levels. Fecal specimens were collected 2×/week and used for the detection of the viral RNA. The fecal specimens were diluted with 10 mM phosphate-buffered saline (PBS) to prepare a 10% (*w*/*v*) stool suspension and shaken at 4 °C for 1 h, then clarified by centrifugation at 10,000× *g* for 30 min. Finally, the suspension was passed through a 0.45 µm membrane filter (Millipore).

The animal experiments were reviewed and approved by the institutional ethics committee and were performed according to the ‘Guides for Animal Experiments at the National Institute of Infectious Diseases, Tokyo, Japan’ under codes 514014 (18 December 2014), 516001 (24 March 2016), and 520003 (24 December 2020). The rabbits were negative for the anti-HEV IgG and IgM antibodies and HEV RNA, and they were individually housed in a Biosafety Level-2 facility.

### 2.3. Extraction and Detection of HEV RNA

We extracted the viral RNA from 200 µL of the serum and fecal samples using a MagNA Pure 96 System (Roche Diagnostics, Mannheim, Germany) with a MagNA Pure 96 DNA and Viral NA Small Volume Kit (Roche Diagnostics) according to the manufacturer’s recommendations. A semi-nested reverse transcription-polymerase chain reaction (RT-PCR) was performed to amplify a portion of the ORF2 genome as described [18] with slight modification. Five microliters of the cDNA was used for the first PCR in 50 µL of the reaction mixture containing an external forward primer, HE044 (5′-CAAGGHTGGCGYTCKGTTGAGAC-3′ [H = A, T, or C; Y = T or C; and K = G or T]) and an external reverse primer, HE040 (5′-CCCTTRTCCTGCTGAGCRTTCTC-3′ [R = A or G]). Two microliters of the first PCR product was used for the nested PCR with HE044 and an internal reverse primer, HE041 (5′-TTMACWGTCRGCTCGCCATTG GC-3′ [M = A or C; W = A or T]). The PCR amplification was conducted under the following conditions: inoculation at 94 °C for 60 s, followed by 35 cycles of 30 s at 94 °C, 30 s at 55 °C, and 75 s at 72 °C, and a final extension at 72 °C for 7 min. The nested PCR products were separated by electrophoresis on 2% agarose gels.

A one-step quantitative real-time RT-PCR (RT-qPCR) was carried out with a 7500 FAST Real-Time PCR System (Applied Biosystems, Foster City, CA, USA) using TaqMan Fast Virus 1-step Master Mix (Applied Biosystems). The RT-qPCR was performed under a protocol of 5 min at 50 °C, 20 s incubation at 95 °C, followed by 40 cycles of 3 s at 95 °C and 30 s at 60 °C using a primer pair of the forward primer 5′-GGTGGTTTCTGGGGTGAC-3′ (nt 5346–5363) and reverse primer 5′-AGGGGTTGGTTGGATGAA-3′ (nt 5393–5415) and the probe 5′-FAM-TGATTCTCAGCCCTTCGC-TAMRA-3′ (nt 5369–5386) [19]. A 10-fold serial dilution of the capped HEV-3 RNA (10^7^ to 10^1^ copies) was used as the standard for the quantification of the viral genome copy numbers. Amplification data were collected and analyzed with Sequence Detector software ver. 1.3 (Applied Biosystems). The detection limit was 10^3^ copies/mL.

### 2.4. Detection of Anti-HEV IgG and IgM Antibodies

Anti-HEV IgG and IgM antibodies were detected by an enzyme-linked immunosorbent assay (ELISA) using virus-like particles (VLPs) as the antigen [20]. The VLPs of HEV-1, HEV-3, HEV-4, and rabbit HEV were produced by the expression of the N-terminal 111 aa-truncated ORF2 protein using recombinant baculoviruses [20,21]. The production of VLPs of HEV-8 is described in the next section. Horseradish peroxidase (HRP)-conjugated goat anti-rabbit IgG-heavy and light-chain antibody (1:5000) (Cappel, Westchester, PA, USA) and HRP-conjugated goat anti-rabbit IgM antibody (1:20,000) (abcam, Tokyo, Japan) were used to detect the rabbit HEV IgG and IgM antibodies, respectively. The cut-off value of the rabbit anti HEV IgG and IgM antibodies were 0.154 and 0.199, respectively [22], and the pre-inoculation rabbit serum was used as negative control.

### 2.5. Expression and Purification of the VLPs of HEV-8

N-terminal 111 aa-truncated ORF2 was amplified by PCR using a plasmid, pUC57-T7HEV-8K, containing the complete genome of HEV-8 (KX387866) as the template [9]. The PCR product was digested with *Bam*HI and *Xba*I and then ligated with a baculovirus transfer vector, pVL1393 (Pharmingen, San Diego, CA, USA) to yield the plasmid pVL1393-G8n111ORF2. An insect cell line, Sf9, was co-transfected with BaculoGold, a linearized wild-type Autographa californica nuclear polyhedrosis virus DNA (BD Biosciences, San Diego, CA, USA) and the transfer plasmid, pVL1393-G8n111ORF2, by a Lipofectin-mediated method as specified by the manufacturer. To achieve large-scale expression, an insect cell line, Tn5, was infected with the recombinant baculoviruses at an m.o.i. of 10, and the cells were cultured in EX-CELL 405 medium (SAFC Biosciences, Lenexa, KS, USA) at 26.5 °C as described [20].

**Figure 1:**
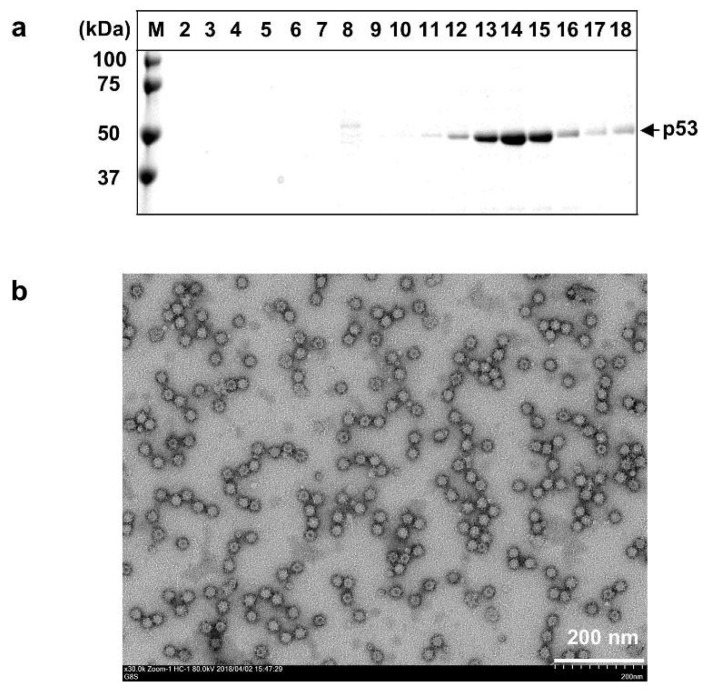
Purification of HEV-8 VLPs. The supernatant of recombinant baculovirus-infected Tn5 cells was collected on day 7 p.i., concentrated by ultracentrifugation, and then purified by CsCl equilibrium density gradient centrifugation. Aliquots from each fraction were analyzed by electrophoresis on 5–20% polyacrylamide gels and stained with Coomassie Brilliant Blue (CBB) (**a**). HEV-8 VLPs with a diameter of approx. 24 nm were observed by a transmission electron microscopy (TEM) using fraction 14 (**b**). Bar: 200 nm. M: molecular weight maker.

The recombinant baculovirus-infected Tn5 cells were harvested on day 7 post-infection (p.i.), and the supernatant was concentrated at 126,000× *g* for 3 h in a Beckman SW32Ti rotor. The resulting pellet was resuspended in EX-CELL 405 medium at 4 °C overnight. For CsCl gradient centrifugation, 4.5 mL of the samples was mixed with 2.1 g of CsCl and centrifuged at 100,000× *g* for 24 h at 10 °C in a Beckman SW55Ti rotor. The gradient was fractionated into 250 µL aliquots; each fraction was diluted with EX-CELL 405 medium and centrifuged for 2 h at 100,000× *g* in a Beckman TLA55 rotor to pelletize the VLPs. Fractions 2 to 18 were analyzed by electrophoresis on 5–20% polyacrylamide gels and stained with Coomassie Brilliant Blue (CBB), a 53-kDa protein (p53) band that processed from N-terminal 111 aa-truncated ORF2 of HEV-8 was primarily distributed in fractions 13–15 with an average density of 1.286 g/cm^3^ (range: 1.282–1.290 g/cm^3^) (Figure 1a). To observe the VLPs we placed the sample on a carbon-coated grid for 45 s, rinsed with distilled water, and stained with a 2% uranyl acetate solution. The grids were observed under a transmission electron microscope (HT7700; Hitachi High Technologies, Tokyo, Japan) at 80 kV. Electron micrographs revealed many spherical particles with a diameter of approx. 24 nm throughout these fractions (Figure 1b).

### 2.6. Liver Enzyme Level

ALT values in the rabbit sera were monitored weekly using a Fuji Dri-Chem Slide GPT/ALT-PIII kit (Fujifilm, Saitama, Japan). The geometric mean titers of ALT over the preinoculation period were defined as normal ALT, and a twofold or greater increase at the peak was considered a sign of hepatitis.

## 3. Results

### 3.1. Rabbits Are Susceptible to HEV-8 Infection

To investigate the susceptibility of rabbits to HEV-5, HEV-7, and HEV-8, we intravenously inoculated rabbits with the culture supernatant of the infected cells containing 1 × 10^7^ copies/mL of the viral RNA. When the HEV-8 was used, the viral RNA was first detected in the feces on day 10 p.i. with 3.44  ×  10^4^ copies/g in rabbit G8Rb-1, on day 7 p.i. with 1.24  ×  10^5^ copies/g in G8Rb-2, and on day 14 p.i. with 5.11 × 10^4^ copies/g in G8Rb-3. After day 14 p.i., no significant increase in the viral RNA was observed. The highest RNA titers were 1.32  ×  10^5^ copies/g on day 28 p.i., 8.54  ×  10^5^ copies/g on day 32 p.i., and 4.80  ×  10^5^ copies/g on day 24 p.i. in rabbits G8Rb-1, G8Rb-2 and G8Rb-3, respectively.

**Figure 2:**
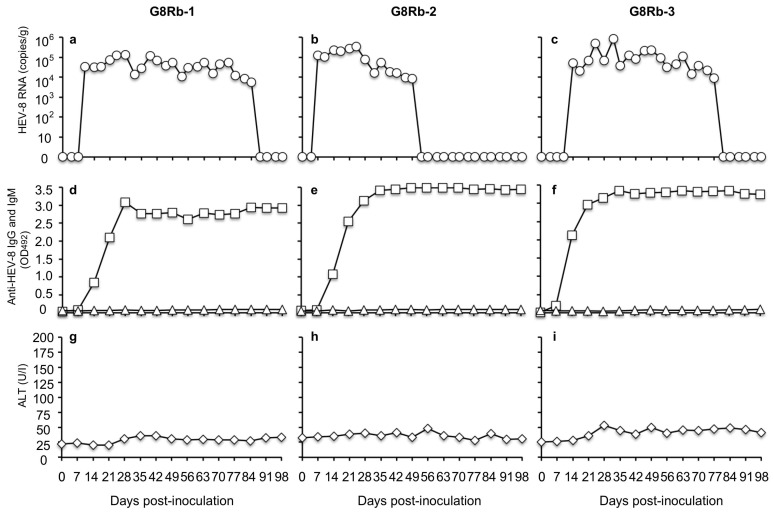
Intravenous infection of rabbits with HEV-8. Three Japanese white rabbits (G8Rb-1, G8Rb-2 and G8Rb-3) were intravenously inoculated with HEV-8, and the kinetics of the viral RNA (○) in the fecal specimens were detected by RT-qPCR (**a**–**c**). Anti-HEV-IgG (☐) and -IgM (△) antibodies (**d**–**f**) were detected using an ELISA with the VLPs of HEV-8 as the antigen. The cut-off value of the rabbit anti HEV IgG and IgM antibodies were 0.154 and 0.199, respectively. ALT levels (◇) were measured (**g**–**i**).

The viral RNA was detected until day 84 p.i. in G8Rb-1, until day 49 p.i. in G8Rb-2, and until day 77 p.i. in G8Rb-3 (Figure 2a–c). To confirm the infection by HEV-8, we amplified a portion of the viral RNA genome by RT-PCR using fecal samples collected on day 24 p.i., and the nucleotide sequence analyses confirmed that the 412 bp of ORF2 were identical to those of the HEV-8 used for the inoculation. The viral RNA was undetectable in the sera collected from these rabbits during the period of this experiment.

The anti-HEV-8 IgG antibodies were first detected on days 14, 14, and 7 p.i. with the OD values of 0.850, 1.069, and 0.199 in rabbits G8Rb-1, G8Rb2, and G8Rb-3, respectively, and the antibody levels quickly peaked on days 28, 35, and 35 p.i. with the OD values of 3.078, 3.418, and 3.416, respectively. Until day 98 p.i., no significant changes in the OD values were observed. Interestingly, neither the anti-HEV-8 IgM antibody responses (Figure 2d–f) nor the ALT elevations were observed until day 98 p.i. (Figure 2g–i). These results indicated that the HEV-8 seemed to cross-infect the rabbits, inducing unique immune responses without any clinical signs.

In contrast, when HEV-5 and HEV-7 were used to inoculate the rabbits, neither the viral RNAs nor the anti-HEV-5 and anti-HEV-7 IgG and IgM antibodies were detected in any of the rabbits. In addition, no elevation of ALT was observed in these rabbits. These results suggested that the rabbits were insusceptible to HEV-5 and HEV-7 infection.

### 3.2. HEV-8-Infected Rabbits Were Not Protected from Challenge by Rabbit HEV

**Figure 3:**
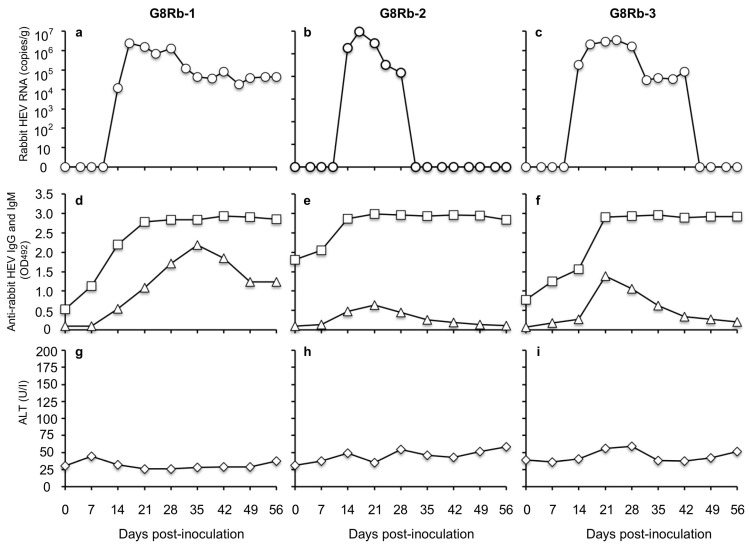
Infection by HEV-8 did not confer inhibitory activity against rabbit HEV infection.The HEV-8-infected rabbits RbG8-1, RbG8-2, RbG8-3 were intravenously inoculated with rabbit HEV. The kinetics of the rabbit HEV RNA (○) in the fecal specimens measured by RT-qPCR (**a**–**c**), anti-rabbit HEV IgG (☐) and IgM (△) antibodies detected by an ELISA with the VLPs of rabbit HEV as the antigen (**d**–**f**), the cut-off value of the rabbit anti HEV IgG and IgM antibodies were 0.154 and 0.199, respectively. ALT levels (◇) were measured (**g**–**i**).

To investigate whether the HEV-8-infected rabbits are protected from other-genotype HEV infection, we inoculated the rabbits (G8Rb-1, G8Rb-2 and G8Rb-3) on day 105 p.i. with rabbit HEV (LC484431) containing 1  ×  10^7^ copies/mL of the RNA. As shown in Figure 3, the rabbit HEV RNA was detected in fecal specimens on day 14 p.i. in rabbits G8Rb-1, G8Rb-2, and G8Rb-3 with the RNA titers of 1.20  ×  10^4^ copies/g, 1.84  ×  10^5^ copies/g, and 1.82  ×  10^5^ copies/g, respectively. The viral RNA reached a peak on day 17 p.i. with 2.45  ×  10^6^ copies/g in G8Rb-1, on day 17 p.i. with 9.44  ×  10^5^ copies/g in G8Rb-2, and on day 24 p.i. with 3.49  ×  10^6^ copies/g in G8Rb-3 (Figure 3a–c). The viral RNA copy numbers then began to decrease, and no viral RNA was detected after day 32 p.i. in G8Rb-2 and after 46 p.i. in G8Rb-3 (Figure 3b,c). In contrast, the viral RNA was continually detected until day 56 in rabbit G8Rb-1 (Figure 3a). To confirm the infection by rabbit HEV, we amplified a portion of the viral RNA genome by RT-PCR using fecal samples collected on day 21 p.i., and the nucleotide sequence analyses confirmed that the 412 bp of ORF2 were identical to those of the rabbit HEV used for the inoculation.

The results of the antibody ELISA based on the VLPs of rabbit HEV indicated that a significant increase of anti-rabbit HEV IgG antibodies occurred in all three rabbits on day 7 p.i. and peaked on day 21 p.i. The anti-rabbit HEV IgM antibodies were detected on day 7 p.i. and peaked on days 35, 21, and 21 p.i., in rabbits G8Rb-1, -2 and -3, respectively, and then gradually decreased (Figure 3d–f). No significant ALT elevation was observed in any of the rabbits (Figure 3g–i). These results demonstrated that the HEV-8-infected rabbits were further infected with rabbit HEV; in other words, the HEV-8-infected rabbits were not protected from infection by rabbit HEV.

### 3.3. Cross-Reactivity of Rabbit Anti HEV-8 IgG Antibodies

The susceptibility of the HEV-8-infected rabbits to the challenge by rabbit HEV raised the question of whether the antigenicity of HEV-8 differs from that of rabbit HEV. We thus examined the reactivity of a series of sera obtained from HEV-8-infected rabbits by conducting the antibody ELISA using the VLPs of rabbit HEV as the antigen. For comparison, the VLPs of HEV-1, HEV-3, and HEV-4 were used.

**Figure 4:**
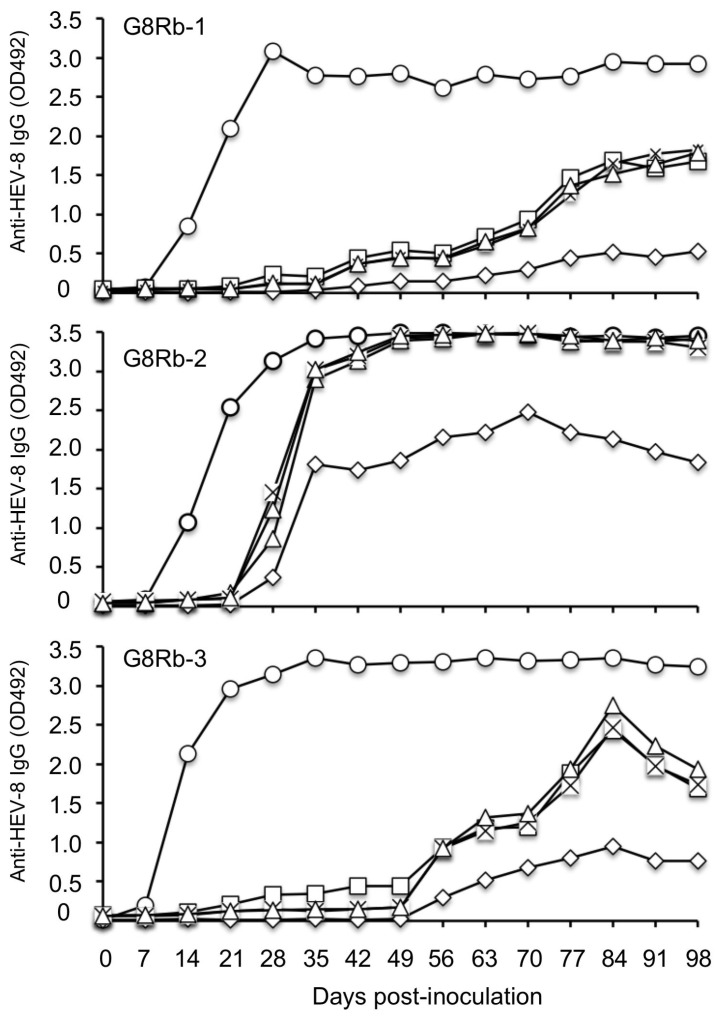
Reactivity between HEV-8-infected rabbit sera and HEV-8, HEV-1, HEV-3, HEV-4, and rabbit HEV. The VLPs derived from HEV-8 (○), HEV-1 (☐), HEV-3 (×), HEV-4 (△), and rabbit HEV (◇) were used to coat 96-well microplates. The reactivity of a series of sera collected from three HEV-8-inoculated rabbits (G8Rb-1, G8Rb-2, and G8Rb-3) with the VLPs was examined by an antibody ELISA to detect anti-rabbit HEV IgG. The cut-off value of the rabbit anti HEV IgG antibody was 0.154. The amino acid sequence identities of the VLPs between HEV-8 (MH410176) and HEV-1, rabbit HEV, HEV-3 and HEV-4 were 93.6%, 93.8%, 95.0% and 95.6%, respectively.

The binding of the anti-HEV-8 IgG antibodies elicited in HEV-8-infected rabbits to the VLPs of HEV-1, HEV-3, HEV-4, and rabbit HEV was examined. As shown in Figure 4, the binding to heterologous VLPs, especially to VLPs of rabbit HEV, was weak in all three rabbits. These results indicated that the antigenicity of HEV-8 differs from that of rabbit HEV.

**Figure 5:**
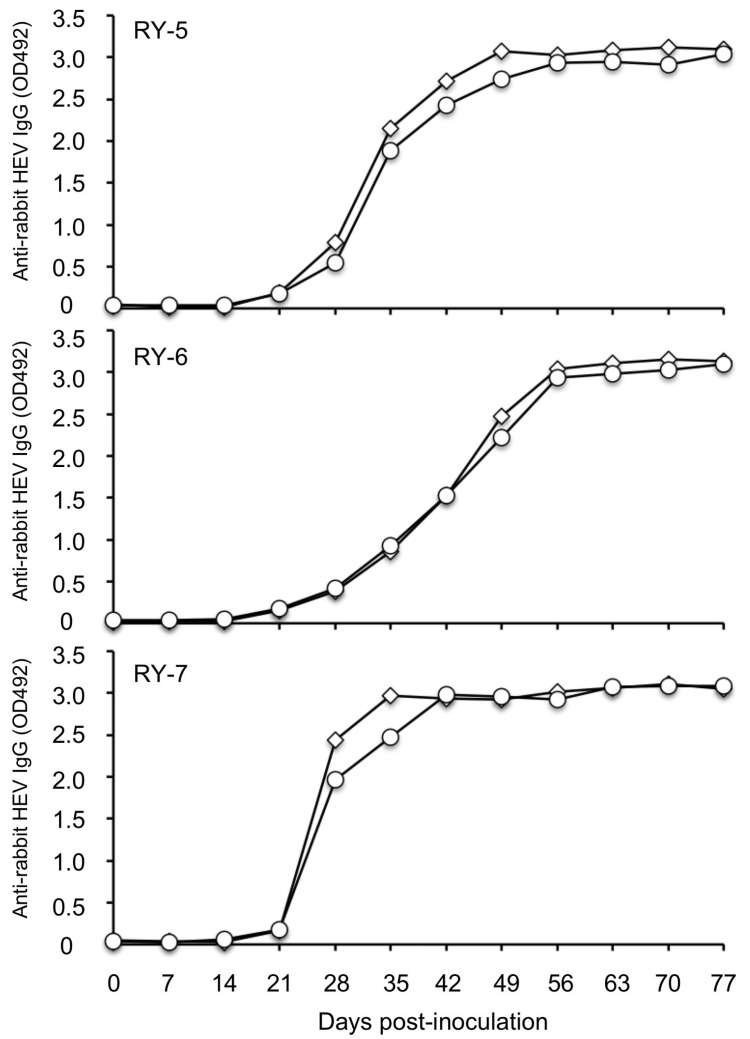
Reactivity between rabbit HEV-infected rabbit sera and HEV-8. The VLPs derived from rabbit HEV (◇) and HEV-8 (○) were used to coat 96-well microplates. The reactivity of a series of the sera collected from three rabbit HEV-inoculated rabbits with the VLPs was examined by an antibody ELISA to detect anti-rabbit HEV IgG. The cut-off value of the rabbit anti HEV IgG antibody was 0.154.

To further investigate the antigenic differences between HEV-8 and rabbit HEV, we examined the binding of the anti-rabbit HEV IgG antibodies elicited in HEV-infected rabbits designated as RY-5, RY-6, and RY-7 [17] to the VLPs of HEV-8 and rabbit HEV. The antibody titers against both VLPs revealed the same OD value in all three rabbits during the infection period (Figure 5), demonstrating that the anti-rabbit HEV antibody had similar reactivity to the VLPs derived from homologous rabbit HEV as well as heterologous HEV-8.

## 4. Discussion

Although no human cases of HEV-5 and HEV-8 have been reported, these novel HEVs carry a risk for zoonotic infection and should be studied in small animal models. Our present results confirmed that rabbits are susceptible to HEV-8 infection and suggested that rabbits could be used as a small animal model of HEV-8 infection (instead of Bactrian camels). However, no elevation of ALT was observed in the HEV-8-infected rabbits, demonstrating that (i) HEV-8 infection did not induce liver damage in the rabbits, and (ii) rabbits are not suitable animals for the study of the pathogenicity of HEV-8. 

Our findings also indicate that rabbits are not susceptible to HEV-5 and HEV-7, suggesting that each HEV genotype has its own host tropism; this feature might be useful for the identification of the receptor(s). Although infectivity of the HEVs was examined by an intravenous inoculation with the viruses recovered from the cell culture supernatants, more experiments are necessary to confirm whether natural HEV-5, HEV-7, and HEV-8 strains can be transmitted to rabbits by oral inoculation.

We observed that during the period of HEV-8 infection, the viral RNA was undetectable in the rabbit serum and the viral RNA titers were <8.54  ×  10^5^ copies/g in the fecal specimens; these values are significantly lower than the value (1.47  ×  10^7^ copies/g) observed when cynomolgus monkeys were inoculated with the same HEV-8 strain [9]. These findings suggest that the HEV-8 replication in rabbits may be limited. In addition, no IgM antibody was detected in any of the three rabbits during the HEV-8 infection, even though the IgM antibody against rabbit HEV was induced in these rabbits by inoculation with rabbit HEV (Figure 3d–f). Anti-HEV-8 IgM antibody was also induced in the cynomolgus monkeys by inoculation with HEV-8; this is a major difference between rabbits and cynomolgus monkeys regarding their susceptibility to HEV-8 [9]. HEV-8 induced a unique immune response in rabbits, and it will be interesting to investigate whether HEV-8 infection fails to induce IgM antibodies or induces a low level IgM response.

It was reported that an intravenous inoculation of 10^6^ copies of HEV-8 derived from a fecal specimen of a Bactrian camel did not induce infection in any of five 3-month-old rabbits of unspecified gender, although an inapparent infection was induced in one of them [16]. In contrast, all three rabbits in the present study, which were 8-month–old females, and were inoculated with HEV-8 containing 10^7^ copies of viral RNA derived from cell culture, did show signs of infection. A future research task will be to determine whether the animal age and gender affected the immune response against HEV, and whether the infectivity of HEV-8 in rabbits is dependent on the viral titers or is related to the source of HEV-8.

We found that the HEV-8-infected rabbits were further infected with rabbit HEV, and the viral RNA copies were decreased or became undetectable as the anti-rabbit HEV IgG antibody increased in the sera, suggesting that rabbit HEV was neutralized by the anti-rabbit HEV antibodies (Figure 3). Anti-rabbit HEV IgM antibodies were clearly detected in all three rabbits, demonstrating that rabbit HEV infection induced a normal immune response in their natural host (Figure 3). Although the HEV-8-infected rabbits were not protected from rabbit HEV infection, the viral titers in their fecal specimens were <3.49  ×  10^6^ copies/g (Figure 3a–c), which is lower than those of the naïve rabbits inoculated with the same rabbit HEV strain (>10^7^ copies/g) [17]. In addition, the rabbit HEV RNA was undetectable in the serum and no elevation of ALT was observed in all three rabbits. These results revealed that the anti-HEV-8 antibodies were likely to partially inhibit rabbit HEV infection.

Our previous studies confirmed that HEV-3, HEV-5, and HEV-7 were neutralized by the anti-HEV-1, -HEV-3, and -HEV-4 antibodies, and that anti-rabbit HEV serum neutralized HEV-1, HEV-3, and HEV-4 ex vivo [6,7,23]. HEV-7-infected cynomolgus monkeys were protected from HEV-3 and HEV-5 infection [6,7]. These results demonstrated that the serotypes of the HEVs in the *Orthohepeviruses* species *A* are similar. The HEV-8-infected rabbits were further infected with rabbit HEV, revealing non-negligible differences in the antigenicity between HEV-8 and rabbit HEV. HEV-8 seemed to lack some epitope(s) that induce the neutralizing antibodies against rabbit HEV. In addition, the reactivity of the anti-HEV-8 IgG antibody to HEV-8 was stronger than those of HEV-1, HEV-3, and HEV-4, and it is thus necessary to determine whether the serotype of HEV-8 differs from those of other genotypes of HEV.

## Data Availability

The sequences of HEV used in the study have been assigned GenBank (accession nos. AB573435, KJ496144, MH410176, LC484431).
